# Prenatal exposure to endocrine disrupting chemicals and the association with behavioural difficulties in 7-year-old children in the SELMA study

**DOI:** 10.1038/s41370-024-00739-x

**Published:** 2024-12-19

**Authors:** Marlene Stratmann, Fatih Özel, Maria Marinopoulou, Christian Lindh, Hannu Kiviranta, Chris Gennings, Carl-Gustaf Bornehag

**Affiliations:** 1 Department of Health Sciences, Karlstad University, Karlstad, Sweden; 2 Department of Organismal Biology, Uppsala University, Uppsala, Sweden; 3 Centre for Women’s Mental Health During the Reproductive Lifespan—Womher, Uppsala University, Uppsala, Sweden; 4 Gillberg Neuropsychiatry Centre, Institute of Neuroscience and Physiology, Sahlgrenska Academy, University of Gothenburg, Gothenburg, Sweden; 5 Child and Adolescent Habilitation, Region Värmland, Karlstad, Sweden; 6 Division of Occupational and Environmental Medicine, Department of Laboratory Medicine, Lund University, Lund, Sweden; 7 Department of Health Security, Finnish Institute for Health and Welfare, 70701 Kuopio, Finland; 8 Department of Environmental Medicine and Public Health, Icahn School of Medicine at Mount Sinai, New York, NY USA

**Keywords:** Prenatal exposure, Mixtures, Endocrine disrupting chemicals, Behavioural difficulties, SDQ, Repeated holdout validation

## Abstract

**Background:**

Endocrine disrupting chemicals (EDCs) can cross the placenta and thereby expose the fetus, which may lead to developmental consequences. It is still unclear which chemicals are of concern regarding neurodevelopment and specifically behaviour, when being exposed to a mixture.

**Objective:**

The objective is to determine associations between prenatal exposure to EDCs and behavioural difficulties. Furthermore, we investigated sex-specific associations and determined chemicals of concern in significant regressions.

**Methods:**

Associations between prenatal exposure to EDCs (both as single compounds and their mixtures) and behavioural outcomes using the Strengths and Difficulties Questionnaire (SDQ) were estimated in 607 mother-child pairs in the Swedish Environmental Longitudinal, Mother and Child, Asthma and Allergy (SELMA) study. Levels for chemical compounds were measured in either urine or serum (median of 10 weeks of gestation). Associations were estimated for the total SDQ score (quasipoisson regression) and a 90th percentile cut-off (logistic regression). Exposure for EDC mixtures (phenols, phthalates, PFAS and persistent chlorinated) was studied using weighted quantile sum (WQS) regression with deciles and with and without repeated holdout validation techniques. The models were adjusted for selected covariates.

**Results:**

The odds for behavioural difficulties increased in girls with higher chemical exposures (OR 1.77, 95% CI 1.67, 1.87) using the full sample and borderline for the validation set (OR 1.31, 95% CI 0.93, 1.85) with 94/100 positive betas in the 100 repeated holdout validations. Chemicals of concern for girls are mostly short-lived chemicals and more specifically plasticizers. No pattern of significant associations was detected for boys.

**Significance:**

There is an indication of increased behavioural difficulties for girls in the SELMA population with higher exposure to mixtures of EDCs. Using the repeated holdout validation techniques, the inference is more stable, reproducible and generalisable. Prenatal exposure to mixtures of environmental chemicals should be considered when assessing the safety of chemicals.

**Impact:**

Growing evidence points towards a “mixture effect” where different environmental chemicals might act jointly where individual compounds may be below a level of concern, but the combination may have an effect on human health. We are constantly exposed to a complicated mixture pattern that is individual for every person as this mixture depends on personal choices of lifestyle, diet and housing to name a few. Our study suggests that prenatal exposure to EDCs might adversely affect the behaviour of children and especially girls. Hence, risk assessment needs to improve and sex-specific mechanisms should be included in assessments.

## Introduction

Endocrine disrupting chemicals (EDCs) are defined as chemicals that can interfere with the hormonal (endocrine) function in humans and animals [1]. Further, EDCs are classified as xenobiotics, as they naturally do not occur in the human body, but can enter through the oral and airway tract, as well as via the skin [2, 3]. According to the European Chemical Agency’s and European Food Safety Authority’s “Guidance for the identification of endocrine disruptors in biocides and pesticides”, an endocrine disruptor is a chemical that shows adverse effects in an intact organism, alters the function of the endocrine system and the adverse effect in the intact organism is a result of the altered function of the endocrine system [4]. Even though some suspected EDCs such as bisphenol A (BPA), and certain phthalates and phenol derivatives have been classified as EDCs according to the REACH legislation (Registration, Evaluation, Authorisation and Restriction of Chemicals), there are still many chemicals that most certainly are EDCs and have not been classified as such and mixture exposures have been disregarded [5]. Phenols, phthalates and perfluorinated substance (PFAS) are common EDCs which can be found in consumer products such as plastic bottles, toys, food cans, building materials, dental sealants, fragrances, synthetic leathers and cleaning products [6, 7]. Additionally, polychlorinated biphenyl (PCB), which is also an EDC has been used in electrical and general building insulation and is often found in food with high fat content due to food chain contamination [8]. Even though many compounds in this study are banned today, they are still present in the environment today due to their persistent properties [8]. It is known that certain EDCs can cross the placenta and thereby expose the fetus [1].

Previous studies on rodents have shown adversities after EDC exposure in early to mid-gestation. Certain EDCs such as BPA and PCBs can affect fetal neurogenesis and gene expression in the brain and that the hypothalamus is particularly affected by oestrogenic EDCs [9, 10]. Further, exposure to triclosan in mice has shown to affect brain development and behaviour including cognition dysfunction, impairments in sociability and increased anxiety-like behaviour [10]. Brain development of mice was even affected when mice pups were exposed prenatally to dibutyl phthalate, leading to delayed neurodevelopment and dark neuron increase in brain cortex [11]. Based on a review by Rebuli and Patisaul, it has been proposed that further studies on EDCs should investigate possible sex differences due to the knowledge gained from animal studies and the different physiology and hormonal action of females and males [9].

During neurogenesis, hormonal signalling is essential not only in fetal development but even in postnatal development [12]. When the fetus is exposed to EDCs, those chemicals are suspected to act by influencing or altering the action of the hormones in the body and thereby the process of neurogenesis and neurodevelopment [12–14]. Hence, exposure to EDCs may affect the hormone signalling and lead to altered behaviour [15, 16], cognitive deficits [17], changed metabolism and growth [18], impaired sexual development and other congenital disorders and malformations [8, 12] with sex-differences between girls and boys.

Although there are policies regarding safe levels of exposure of EDCs, these regard single chemicals and not exposure to mixtures of EDCs, which correspond to real-life exposures where humans always are exposed to a huge number of compounds [2]. While it is important to study single chemical exposures as previous studies have done [12, 13], mixture approaches should be studied in more detail, as there might be a joint action between chemicals where the adversity is not present in single compound analysis but can be detected in mixture analyses; i.e., the something from nothing phenomenon [19, 20].

The aim of this study is to assess the associations between prenatal exposure to 26 EDCs and adverse behavioural outcomes in children, and to examine if such associations are different between girls and boys. When there are significant associations in boys and/or girls, chemicals of concern will be identified to identify the main contributors to the association.

## Methods

### Study population

The Swedish Environmental Longitudinal Mother and Child, Asthma and Allergy (SELMA) pregnancy cohort included pregnant women at their first antenatal care centre visit (median 10 weeks) in Värmland county, Sweden [21]. The recruitment period started in September 2007 and ended in March 2010, where 6658 women were invited to participate. Exclusion criteria included not understanding written Swedish as data were collected through self-administered questionnaires and not living in, or moving outside of the county of Värmland and having surpassed gestational week 22 [21]. This resulted in 2582 women that agreed to participate (39%). The recruitment process has previously been described in more detail and a visual flow chart of the study population can be seen in Supplementary Fig. 1 [21]. Exposure and baseline characteristic data were taken at enrolment and at birth. Outcome variables were measured at follow-up when the child was approximately 7.5 years old. The study population consists of 607 mother and child pairs. Participating families provided written consent and the study was ethically approved by the Regional Ethic Review Board in Uppsala, Sweden (Dnr: 2007/062 and Dnr: 2015/177). All methods were performed in accordance with the relevant guidelines and regulations.

### Prenatal EDC exposure

First morning void urine was sampled the day after the first antenatal care clinic visit (median 10 weeks) and then stored at −20 °C at the Laboratory of Occupational and Environmental Medicine (OEM) at Lund University, Lund, Sweden. Liquid chromatography tandem mass spectrometry (LC-MS/MS; QTRAP 5500, AB Sciex, Framingham, MA, USA) was used to quantify 24 nonpersistent urinary analytes (Triclosan, BPA, BPF, BPS, MEP, MBP, MBzP, MEHP, MEHHP, MCMHP, MEOHP, MECPP, DEHP, MHiNP, MOiNP, MCiOP, DINP, MHiDP, MCiNP, MOiNCH, DPHP, TCP, PBA and 2OHPH) [21], according to the method described by Gyllenhammar et al. [22]. In urine analyses, the sum of di-(2-ethylhexyl) phthalate (DEHP) and diisononyl phthalate (DINP) metabolites were calculated and used for statistical analysis, instead of the single metabolites. Further, machine read values were used for urinary compounds, if the values were below the limit of detection (LOD). Furthermore, for the mixture analysis we included 26 chemicals, as seen in Table 2 and summarised where appropriate and have included limits of detection and limits of quantification.

In serum, six per- and polyfluoroalkyl substances (PFAS) (PFOA, PFOS, PFNA, PFDA, PFUnDA, PFHxs) and cotinine, a biomarker of tobacco exposure, was analysed at OEM according to a modified method described in Norén et al. and Axelsson et al. [23, 24], also at the first antenatal care clinic visit (median 10 weeks). Briefly, serum proteins were precipitated with acetonitrile and labelled internal standards for all compounds were added. The supernatant was centrifuged before quantification using LC-MS/MS. The laboratory participates in G-EQUAS interlaboratory exercises for analysis of many of the compounds in urine and serum.

Serum was further analysed for 14 persistent compounds and persistent organic pollutants at the National Institute for Health and Welfare, Finland. Ethanol and C^13^-labelled internal standards in toluene were mixed with 200 μl samples to precipitate proteins and equilibrate standards. Dichloromethane-hexane (1:4), followed by activated silica were added for extraction and separation. The dichloromethane-hexane was poured over a solid-phase extraction cartridge (SPE cartridge) with 10% AgNO_3_ impregnated silica and a mixture of Na_2_SO_4_ and silica. Analytes were quantified using gas chromatography—high triple quadrupole mass spectrometry (Agilent 7010 GC-MS/MS system (Wilmington, DE, USA), GC column DB-5MS UI (J&W Scientific, 20 m, ID 0.18 mm, 0.18 μm)). Similar to Tanner et al. [17], Svensson et al. [18], and Mitro et al. [25], ten PCB analytes were summed to PCB and Dichlorodiphenyltrichloroethane (DDT) and its metabolite dichlorodiphenyldichloroethylene (DDE) were summed to DDT.

For levels below quantification in serum compounds, the values were substituted with the level of quantification divided by the square root of 2 (LOQ/$\surd 2$). For the mixture analysis we included 26 chemicals, as seen in Table 2 and summarised where appropriate and have included limits of detection and limits of quantification. A correlation matrix for the included chemicals is shown in Supplementary Fig. 2.

At the time of data collection (2007–2010) these 26 chemicals were suspected to be chemicals of concern and many were expected or proven to be endocrine disrupting which is why these specific chemicals were chosen to be analysed in this cohort.

### Behaviour

Children’s behaviour was measured at 7.5 years (standard deviation (SD) 0.3 years) using the parental version of the Strengths and Difficulties Questionnaire (SDQ) [26]. This brief questionnaire can be used to screen for behavioural difficulties [27], and has been validated for use in a Swedish setting [28]. This instrument includes 25 questions that are scored on a 3-item scale (not true, somewhat true and certainly true). The SDQ can be used to compute the total score (0–40), which is the sum of the four difficulty subscores (each 0–10): emotional, peer- relationship, conduct, hyperactivity/ inattention difficulties. Four of the five subscores are used as indicator for difficulties, with higher scores indicating more problems in all scales except the prosocial subscale, which is a strength sub score and hence, not included in the total SDQ score [26]. Due to a limited sample size and the aim of the study, we did not include subscore analyses. Further, a 90th percentile approximation was used to dichotomise the total score and to analyse potential clinically relevant scores and was eventually set to 10.3% corresponding to a score of 11 and higher instead of the proposed score of 13 and higher [26, 29]. A cut-off at exactly the 90th percentile, as suggested by Goodman et al. [26], was not possible due to the nature of the data distribution. Finally, we used both the total score and the 90th percentile approximation in order to quantify a general change in behavioural difficulties as well as potential clinically relevant behavioural difficulties. The distribution of the SDQ score can be seen in Supplementary Fig. 3.

### Covariates

Covariates were chosen a priori using a directed acyclic graph (DAG) [30], which is shown in the Supplementary Material (Supplementary Fig. 4). Parity is an important risk factor which is also related to the “dumping” effect of maternal stored toxins in breast milk to the first born. We constructed a parity-residual variable based on a linear regression model relating the serum compounds to parity to adjust out the dumping effect. By using the residuals of the regression model on parity instead of the unchanged parity variable, we had a smaller variance which makes the model more accurate. Further, parity has been shown to be a risk factor for pregnancy complications and adverse pregnancy outcomes [31], and by using the parity-residual instead of the unchanged parity variable we were able to hold the effect of the EDCs constant over parity instead of adjusting for potential pregnancy complications and adverse outcomes only. Maternal variables included were the adjusted parity variable, tobacco exposure which included maternal current smoking at the first antenatal care clinic visit, or exposure to tobacco from any person in the household or serum cotinine levels ≥0.2 ng/mL (yes, no), graduated university (yes, no), age at birth (continuous), body mass index (BMI) (continuous) and urinary creatinine levels to adjust for urinary dilution of short-lived compounds. The variables included for the children were age at outcome assessment (continuous) and sex (girls, boys). All covariates except cotinine levels were collected by self-administered questionnaire and BMI, parity and maternal age at birth were centred at the mean. Gestational age at birth and birthweight were not included as covariates as these might be mediators in the association between prenatal exposures and behaviour, as shown in the hypothesised DAG.

### Statistical analyses

Descriptive statistics were used to describe central tendencies or proportions of the population characteristics. EDC exposure variables were natural log transformed and geometric means and geometric standard deviations were calculated for the description of the exposure.

Due to the right skewed distribution of the outcome (Supplementary Fig. 3) quasipoisson regression models were deemed most suitable to estimate associations. Quasipoisson regression models were used to estimate the association (*β*, 95% confidence interval [CI]) between the prenatal EDC exposure (log10-transformed for statistical inference in all models to decrease the potential for influential observations, centred and scaled) and the total SDQ score (total score).

Logistic regression (Odds ratios (OR), 95% CI) was used to estimate the association between prenatal EDC exposure and a dichotomised SDQ score using a 90th percentile approximation, i.e., a cut-off of 11 to examine clinical relevance.

Finally, weighted quantile sum (WQS) regression models were used to identify mixtures and chemicals of concern for the outcome as a metric for the joint action of the components in a mixture effect, as suggested by Carrico et al. [32]. To strengthen the generalisability of the results, we extended the WQS regression with 100 repeated holdout validations, which randomly partitions the data into training (40%) and validation sets (60%) and estimated weights using 100 bootstraps. This provides results that are more stable to extreme values, as described by Tanner et al. [33]. Interaction by exposure levels and sex were examined. As biological mechanisms due to hormonal signalling might be different for boys and girls and sex-specific association have been found in humans and rodents, we conducted sex-stratified analyses [3, 9]. The WQS regression R package (gWQS) has a function to estimate stratified interaction models where estimates are calculated using an interaction term between the mixture and sex, while the specific weights are calculated in a stratified manner, i.e. stratified for girls and boys. Coefficients were restricted to the positive direction, as we were interested in positive (adverse) associations (i.e., increased exposure and higher score on the SDQ scale) rather than the opposite. The WQS regression was conducted both on a continuous (quasipoisson regression) and dichotomised (logistic regression) scale as in the case of single compounds described above. WQS regression disregards the units the chemicals were measured in, as all the included compounds are decile scored to accommodate interpretation of the weights in a weighted index. The threshold for chemicals of concern is estimated by dividing 100% by the 26 included chemicals to provide a value of equal weighting as suggested by Tanner et al. and Carrico et al. [32, 33]. This provides a threshold of 3.8%. To increase the generalisability of the WQS regression models we used 100 repeated holdout validations, as suggested by Tanner et al. [33]. We followed the Busgang criteria to characterise chemicals as *probable* or *possible* contributors; i.e., chemicals with 90% of repetitions above the chemical of concern threshold were defined as being probable contributors to the mixture effect and those with fifty to *<*90% of repetitions above threshold were defined as possible contributors [34, 35]. Non-linearity of mixture models was assessed and can be seen in Supplementary Figs. 5 and 6.

In sensitivity analyses, we explored changes in estimates by gestational age (*n* = 597) and birthweight (*n* = 597) by including each term in quasipoisson and logistic WQS regression analyses. Further, we included maternal IQ as a potential confounder (*n* = 597). Maternal IQ was measured using the shortened Ravens Standard Progressive Matrices [36, 37]. Finally, we also added gestational age, birthweight and maternal IQ into both quasipoisson and logistic WQS regression models (*n* = 597). In addition to analysis for single compounds that were centred and scaled in the main analysis, we explored associations when single chemicals were deciled to compare to the WQS regression results. The analysis for single compounds was corrected for multiple testing by using the false discovery rate. Chemicals above the threshold in the figures for single compounds are significant even after correction. Further, as the mixture analysis uses deciles for the weighted index, we explored the associations using quartiles instead to see if associations differ.

All analysis were performed using the statistical software R version 4.1.2. For access to any code used in this analysis, please refer to the corresponding author.

## Results

### Descriptive statistics

The population characteristics stratified by child’s sex can be seen in Table 1. Slightly less than half of the mothers to girls (46.7%) and boys (48.2%) were nulliparous. More than half of the mothers graduated university (65.6% for girls and 65.6% for boys) and the mothers were on average 31.0 years old for both girls and boys. A total of 10.6% of mothers to girls had an indication for tobacco exposure, whereas it was 8.5% for mothers of boys. The mean age at outcome assessment was 7.5 years for both girls and boys. A mean for total SDQ score of 5.3 and 5.7 were found for girls and boys, respectively. Less girls had a score above the 90th percentile approximation (8.6%) than boys (11.1%).

**Table 1:** Population characteristics stratified by sex (*n* = 607).

	Girls		Boys		Total	
	*n* = 302		*n* = 305		*n* = 607	
Maternal characteristics						
Parity (*n*, %)						
>1 child	161	53.3%	158	51.8%	319	52.6%
1st child	141	46.7%	147	48.2%	288	47.4%
Tobacco exposure^a^ (*n*, %)						
No	270	89.4%	279	91.5%	549	90.4%
Yes	32	10.6%	26	8.5%	58	9.6%
Graduated university (*n*, %)						
No	104	34.4%	105	34.4%	209	34.4%
Yes	198	65.6%	200	65.6%	398	65.6%
Age at birth (mean, SD)	31.0	4.6	31.0	4.3	31.0	4.4
BMI (mean, SD)	24.7	4.5	24.4	4.1	24.5	4.3
Child characteristics						
Age at outcome assessment (mean, SD)	7.5	0.3	7.5	0.3	7.5	0.3
SDQ score (median, IQR)	5.3	4.3	5.7	4.6	5.5	4.4
Above the 90th percentile approximation for SDQ score^b^ (*n*, %)	26	8.6%	34	11.1%	60	9.9%

^a^Tobacco exposure based on questionnaire if the mother, father or another person in the household smoked or serum cotinine levels ≥0.2 ng/mL.

^b^Corresponds to a score of 11 and higher.

Differences in population characteristics for the included (*n* = 607) and the excluded (*n* = 354) study population can be seen in Supplementary Table 1. Included mothers were more likely to have graduated university and to have a lower BMI. Their children were slightly younger with a lower SDQ score.

Crude chemical concentrations of the 26 EDCs are presented in Table 2. For the bisphenols, BPA has the highest GM with 1.50 ng/mL and bisphenols S (BPS) the lowest with a GM of 0.07 ng/mL. For the phthalates, monobutyl phthalate (MBP) had the highest GM of 68.98 ng/mL, followed by monoethyl phthalate (MEP) with 64.72 ng/mL. 2-4-methyl-7-oxyooctyl-oxycarbonyl-cyclohexane carboxylic acid (MOiNCH) and monocarboxyisononyl phthalate (MCiNP) had the lowest GM with 0.30 and 0.69 ng/mL respectively. Regarding PFAS, perfluorooctane sulfonate (PFOS) had the highest concentration with 5.58 ng/mL and perfluoroundecanoic acid (PFUnDA) the lowest with 0.22 ng/mL. Among the persistent chlorinated compounds, dichlorodiphenyltrichloroethane (DDT) had the highest GM with a value of 0.19 ng/mL, whereas trans-nonachlor (Nonachlor) had the lowest GM with 0.01 ng/mL.

**Table 2:** Level of chemical concentrations (ng/mL) measured at first antenatal clinic visit, median 10 weeks of gestation (*n* = 607).

		LOD/LOQ	≥LOD/LOQ	GM (GSD)	Included in WQS analysis
Matrix	Metabolites				
Urine	Phenols				
	Triclosan	0.100	100.0%	1.33 (9.98)	X
	BPA	0.050	100%	1.50 (2.41)	X
	BPF	0.024	100%	0.16 (5.24)	X
	BPS	0.009	100%	0.07 (3.00)	X
	Phthalates				
	MEP	0.010	100%	64.72 (2.98)	X
	MBP	0.100	100%	68.98 (2.22)	X
	MBzP	0.040	100%	15.16 (2.96)	X
	MEHP	0.100	100%		
	MEHHP	0.020	100%		
	MCMHP	0.068	100%		
	MEOHP	0.030	100%		
	MECPP	0.020	100%		
	∑ DEHP	–	–	56.35 (2.42)	X
	MHiNP	0.020	100%		
	MOiNP	0.010	100%		
	MCiOP	0.020	100%		
	∑ DINP	–	–	20.20 (2.98)	X
	MHiDP	0.031	100%	1.26 (2.73)	X
	MCiNP	0.031	100%	0.69 (2.32)	X
	MOiNCH	0.023	98.8%	0.30 (3.99)	X
	DPHP	0.042	100%	1.35 (2.56)	X
	Other short-lived chemicals				
	TCP	0.035	100%	1.25 (2.49)	X
	PBA	0.017	99.5%	0.16 (2.68)	X
	2OHPH	0.003	100%	0.20 (2.32)	X
Serum	Perfluoro-alkyl substances (PFAS)				
	PFOA	0.020	100%	1.63 (1.74)	X
	PFOS	0.060	100%	5.58 (1.71)	X
	PFNA	0.010	100%	0.55 (1.66)	X
	PFDA	0.020	100%	0.27 (1.59)	X
	PFUnDA	0.020	99.8%	0.22 (1.80)	X
	PFHxS	0.030	100%	1.33 (1.76)	X
	Persistent chlorinated				
	HCB	0.005	100%	0.05 (1.37)	X
	Nonachlor	0.005	100%	0.01 (1.83)	X
	DDTa	0.015	100%	–	
	DDE	0.040		–	
	∑ DDT	–	–	0.20 (1.81)	X
	PCB 74	0.005	76.7%	–	
	PCB 99	0.005	84.1%	–	
	PCB 118	0.005	100%	–	
	PCB 138	0.005	100%	–	
	PCB 153	0.005	100%	–	
	PCB 156	0.005	93.1%	–	
	PCB 170	0.005	100%	–	
	PCB 180	0.005	100%	–	
	PCB 183	0.005	79.3%	–	
	PCB 187	0.005	100%	–	
	∑ PCB	–	–	0.41 (1.59)	X

*LOD* limit of detection for urinary metabolites, *LOQ* limit of quantification for serum metabolites, *GM* geometric mean, *GSD* geometric standard deviation, *2OHPH* 2-hydroxyphenanthrene, *BPA* bisphenol A, *BPF* bisphenol F, *BPS* bisphenol S, *DEHP* di-(2-ethylhexyl) phthalate, *DDE* dichlorodiphenyldichloroethylene, *DDT* dichlorodiphenyltrichloroethane, *DINP* diisononyl phthalate, *DPHP* diphenylphosphate, *HCB* hexachlorobenzene, *MBP* monobutyl phthalate, *MBzP* monobenzyl phthalate, *MCiOP* mono(carboxy-iso-octyl) phthalate, *MCiNP* monocarboxyisononyl phthalate, *MCMHP* mono(2-carboxymethylhexyl) phthalate, *MECPP* mono(2-ethyl-5-carboxypentyl) phthalate, *MEHP* mono(2-ethylhexyl) phthalate, *MEHHP* mono(2-ethyl-5-hydroxyhexyl) phthalate, *MEOHP* mono(2-ethyl-5-oxohexyl) phthalate, *MEP* monoethyl phthalate, *MHiDP* monohydroxyisodecyl phthalate, *MHiNP* mono(hydroxyisononyl) phthalate, *MOiNP* mono(oxoisononyl) phthalate, *MOiNCH* 2-4-methyl-7-oxyooctyl-oxycarbonyl-cyclohexane carboxylic acid, *PBA* 3-phenoxybenzoic acid, *Nonachlor* trans-nonachlor, *PCB* polychlorinated biphenyl, *PFAS* per- and polyfluoroalkyl substances, *PFDA* perfluorodecanoic acid, *PFHxS* perfluorohexane sulfonic acid, *PFNA* perfluorononanoic acid, *PFOA* perfluorooctanoic acid, *PFOS* perfluorooctane sulfonate, *PFUnDA* perfluoroundecanoic acid, *TCP* 3,5,6-trichloro-2-pyridinol.

### Mixture analysis

The adjusted association between prenatal EDC mixtures (WQS-scores) and behavioural outcomes are shown in Table 3.

**Table 3:** Associations between the mixture of 26 chemicals and continuous SDQ score estimated by quasipoisson regression using betas and 95% CI and the dichotomised SDQ score with a 90th percentile cut-off approximation estimated by logistic regression using OR and 95% CI (*n* = 607).

	Adjusted^a^			Stratified^b^						Interaction^c^
	All children (*n* = 607)			Girls (*n* = 302)			Boys (*n* = 305)			
	Estimate	95% CI	Positive beta^d^	Estimate	95% CI	Positive beta^d^	Estimate	95% CI	Positive beta^d^	
Full sample^e^										
Quasipoisson WQS (*β*)	0.09	0.04, 0.15		0.12	−0.02, 0.27		0.09	0.02, 0.16		*p* = 0.36
Logistic WQS (OR)	1.35	1.05, 1.74		1.77	1.67, 1.87		1.33	0.99, 1.78		*p* = 0.14
Validation^f^										
Quasipoisson WQS (*β*)	0.03	−0.03, 0.08	86/100	0.03	−0.03, 0.10	87/100	−0.01	−0.08, 0.06	40/100	*p* = 0.25
Logistic WQS (OR)	0.97	0.73, 1.29	48/100	1.31	0.93, 1.85	94/100	0.87	0.62, 1.26	21/100	*p* = 0.09

^a^Adjusted for parity, maternal tobacco exposure, maternal education, maternal age, maternal BMI, child sex and child’s age at outcome assessment and creatinine.

^b^Adjusted for parity, maternal tobacco exposure, maternal education, maternal age, maternal BMI and child’s age at outcome assessment and creatinine.

^c^Interaction between the mixture of EDCs and sex.

^d^Number of positive betas from 100 repeated holdout validations.

^e^WQS regression using all 607 children in a single quasipoisson or logistic model.

^f^100 repeated holdout validations using 40% of the data as training and 60% as validation data.

Significant associations can be seen in the adjusted quasipoisson WQS model for all children (*β* 0.09, 95% CI 0.04, 0.15 [for one unit increase in the exposure deciles, the total SDQ score increases by 0.09 points]). In analyses stratified by sex there were no significant increases for girls (*β* 0.12, 95% CI −0.02, 0.27), but there were for boys (*β* 0.09, 95% CI 0.02, 0.16). The adjusted logistic WQS model (using the cut-off ≥11) showed significantly higher odds for having a total SDQ score above the cut-off for all children (OR 1.35, 95% CI 1.05, 1.74 [the odds of having a score above the cut-off were 1.35 times higher in decile n, compared to decile *n*−1]). In the WQS sex-stratified interaction regression models, the girls showed significantly increased ORs (OR 1.77, 95% CI 1.67, 1.87) whereas the boys show borderline significance (OR 1.33, 95% CI 0.99, 1.78).

In the same models, we used 100 repeated holdout validations and yielded no statistically significant results in any of the WQS regressions, as the estimates were attenuated. However, girls showed 87/100 positive betas for quasipoisson and 94/100 positive betas for logistic WQS regression models, indicating a borderline association, as also the logistic regression models using 100 repeated holdout validations were close to significance (OR 1.31, 95% CI 0.93, 1.85).

### Chemicals of concern

**Fig. 1:**
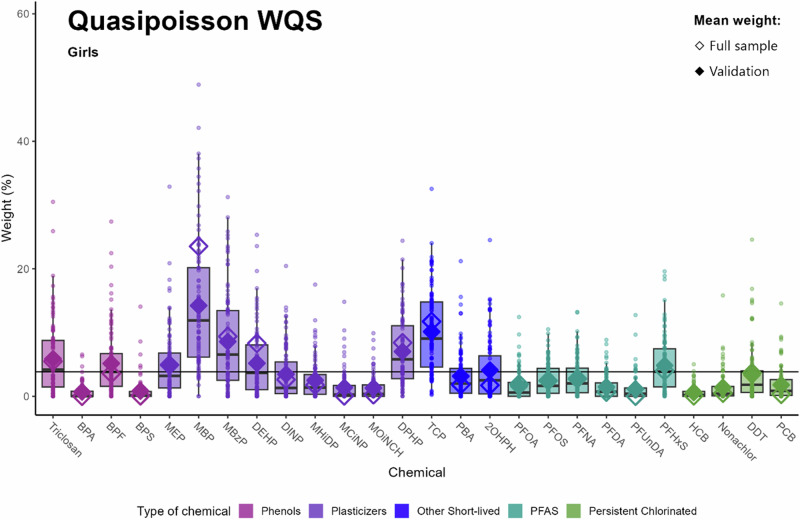
Identification of chemicals of concern and uncertainty using traditional full sample vs. repeated holdout validation: adjusted quasipoisson WQS for girls (*n* = 302). Data points indicate weights for each of the 100 holdouts. Box plots show 25th, 50th and 75th percentiles and whiskers show 10th and 90th percentiles of weights for the 100 holdouts. Closed diamonds show mean weights for the 100 holdouts and open diamonds show the mean weight of the full sample analysis. The black line at 3.84% indicates the threshold of chemicals of concern. Models were adjusted parity, maternal tobacco exposure, maternal education, maternal age, maternal BMI, child sex and child’s age at outcome assessment and creatinine.

Figure 1 shows the weights for all 26 chemicals in girls using quasipoisson WQS regressions. The empty diamonds refer to the chemicals of concern in the full sample analysis while the filled diamonds correspond to the chemicals of concern in the validation analysis. Focusing on the weights in the validation set in descending order, MBP, TCP, MBzP, DPHP, Triclosan, DEHP, BPF, MEP, PFHxS and 2OHPH were of concern. In the following sections, all chemicals are mentioned in descending order.

**Fig. 2:**
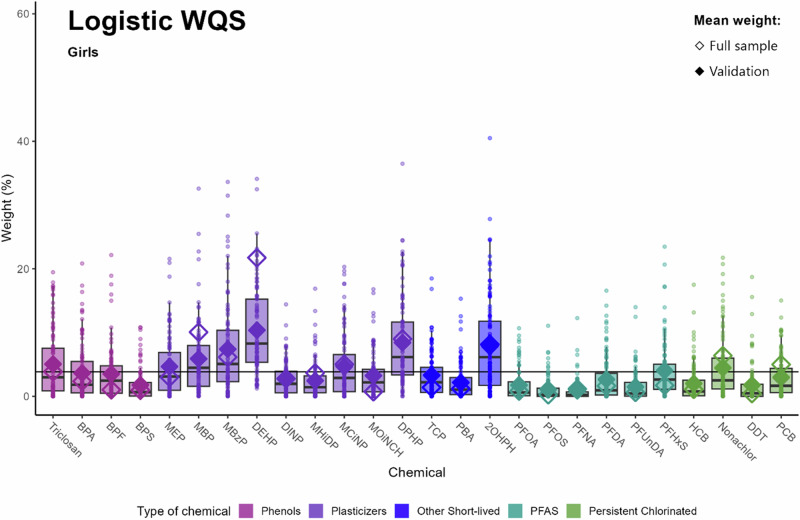
Identification of chemicals of concern and uncertainty using traditional full sample vs. repeated holdout validation: adjusted logistic WQS for girls (*n* = 302). Data points indicate weights for each of the 100 holdouts. Box plots show 25th, 50th and 75th percentiles and whiskers show 10th and 90th percentiles of weights for the 100 holdouts. Closed diamonds show mean weights for the 100 holdouts and open diamonds show the mean weight of the full sample analysis. The black line at 3.84% indicates the threshold of chemicals of concern. Models were adjusted parity, maternal tobacco exposure, maternal education, maternal age, maternal BMI, child sex and child’s age at outcome assessment and creatinine.

In Fig. 2, the chemicals of concern for girls in logistic regression analysis are shown. Ten chemicals can be identified as chemicals of concern: DEHP, DPHP, 2OHPH, MBzP, MBP, Triclosan, MCiNP, MEP, Nonachlor and PFHxS.

Comparing Figs. 1 and 2, eight chemicals could be identified in both the quasipoisson and logistic regression analysis (Triclosan, MEP, MBP, MBzP, DEHP, DPHP, 2OHPH and PFHxS), while BPF and TCP were only identified in the quasipoisson WQS regression and MCiNP and Nonachlor only in the logistic WQS regression.

In Supplementary Figs. 7 and 8 the same results are shown for boys. As these were non-significant, we show them for completeness. A summary of chemicals of concern can be seen in Supplementary Table 2. There, each chemical that was of concern at least 50 out of 100 repeated holdout validations is denoted as a possible contributor to the mixture effect that is shown in Table 3.

### Sensitivity analysis

The association between single compounds and SDQ are presented in Supplementary Figs. 9–14. In Supplementary Fig. 9, the multiple testing corrected estimates are shown for single chemicals and the continuous SDQ score for all children and then for girls (Supplementary Fig. 10) and boys (Supplementary Fig. 11). Only in Supplementary Fig. 10 for the girls, MBP is significant after correcting with the false discovery rate, which indicates that MBP is highly significant and important potentially even in single chemical analysis.

In Supplementary Figs. 12–14, the association between single compounds and the dichotomous SDQ score is shown using logistic regression analysis and correcting for multiple testing. None of the estimates remained significant after correction.

Supplementary Table 4 shows quasipoisson and logistic WQS regression models that were further adjusted for birthweight or gestational age in order to estimate a potential mediating effect and maternal IQ as a possible confounder. Further, we included all covariates included in the main model and both potential mediators and maternal IQ as a possible confounder in WQS regression models. The model including gestational age and maternal IQ are very similar to our main analyses (*β* 0.09, 95% CI 0.04, 0.14 and *β* 0.09, 95% CI 0.04, 0.15, respectively), whereas the model including birthweight and the model including all three variables show decreased estimates (*β* −0.02, 95% CI −0.09, 0.04 and *β* −0.02, 95% CI −0.09, 0.04, respectively).

The same pattern can be seen in the logistic regression models with the models including gestational age or maternal IQ having similar estimates (OR 1.39, 95% CI 1.07, 1.81 and OR 1.39, 95% CI 1.08, 1.81, respectively) and the models including birthweight and all three variables are attenuated (OR 1.07, 95% CI 0.79, 1.44 and OR 1.07, 95% CI 0.80, 1.44).

In Supplementary Table 5, the quasipoisson and logistic WQS regression models using deciles are compared to quartiles. The pattern of significance is the same whereas the estimates are larger when using quartiles compared to deciles and the confidence intervals are wider.

## Discussion

We found an overall increase in behavioural difficulties at 7 years in connection with exposure to mixtures of chemicals. Especially in girls there seems to be an increase in behavioural difficulties in the specific SELMA population but also in the validation analysis which means that there is potential risk for all girls. This association was mainly driven by eight chemicals of which six were plasticizers (MEP, MBP, MBzP, DEHP, DPHP, 2OHPH), one phenol (Triclosan) and one PFAS (PFHxS). Thus, even exposures to chemicals that are considered to have a short half-life like the mentioned plasticizers and the phenol compound, are potentially harmful.

### Mixture of chemicals and chemicals of concern

The aim of the study was to assess the association between prenatal mixtures of 26 EDCs and behavioural difficulties in children. These EDCs were selected for analysis roughly 15 years ago based on their purported adverse effects on human development, including neurodevelopment. We do not anticipate that any of these chemicals would be beneficial to neurodevelopment. As such, we focused our hypothesis and related analysis strategy in the positive, i.e., adverse, direction of the outcome resulting in a more powerful statistical test. Using WQS regression as a supervised strategy for characterising the mixture effect of the EDCs, and more specifically, in a stratified interaction model, we were able to detect at least a borderline significant positive (adverse) association for girls. This contrasts with the single chemical analyses where only MBP passed the multiple testing correction for an adverse effect. None of the other single chemicals were significant alone.

Our study is mostly in line with previously conducted studies on the prenatal exposure to EDC mixtures and behaviour in children. Guilbert et al. found an increase in problem behaviour in the French mother and child cohort SEPAGES, including 416 2-year-old children using similar chemicals (phenols and phthalates) [16]. In girls, the internalising and externalising scores were increased (2.47 points, 95% CI 0.60, 4.33 and 3.67 points, 95% CI 1.24, 6.10, respectively) but not in boys [16]. Even though the outcome was assessed using the Child Behaviour Checklist (CBCL) and the samples for exposure classification were collected multiple times in the French cohort, similarities in the results cannot be disregarded. Three of the main contributors (Triclosan, MEP, MBzP) in the SEPAGES cohort for girls could also be found in our sample for girls [16]. Even though the compared study used specific gravity to adjust chemical concentrations for urine dilution and they collected more urine samples over different time points in pregnancy, we were able to replicate the finding of MBzP being a chemical of concern.

Further, a study from the USA conducted by Day et al. the TIDES study (*n* = 501), also found an increase in problem behaviour, in connection to increased exposure in the phthalate mixture, that was evident in both sexes after stratification [38]. Chemicals of concern for girls were MCPP, MEP, MBzP and MBP [38]. Similar to our study, MEP, MBzP and MBP seems to be a chemical of concern as the TIDES study by Day et al. and the SEPAGES cohort by Guilbert et al. found similar results [16, 38].

Some studies showed different chemicals of concern of which not all could be found in the current study. Different chemicals were chosen to be included in the mixture analysis, but differences where the same chemicals were included could lie in the multiple assessment of the prenatal exposure in other studies [15, 16, 38, 39] to approximate the real chemical concentration in the maternal urine and serum. Differences could also be due to the choice of different questionnaires to quantify the problem behaviours. These included the CBCL [16, 40], the Behavioral Assessment System for Children, 2nd edition [38, 39], the Social Responsiveness Scale, 2nd edition (SRS-2) [38] and the Conner’s Parent Rating Scale-Revised: Long Form [40]. One study used clinically diagnosed ASD as well as the Autism Diagnostic Observation Scale and Mullen Scales of Early Learning [15]. While all mentioned questionnaires capture problem behaviours, the SDQ captures a broader range of different behaviour and is hence less comprehensible in comparison to the other above-mentioned instruments [41, 42]. Further, the SRS-2 mainly focuses on ASD-related difficulties and did not match our aim [41] and the CBCL covers a broader range of behaviours, but the CBCL has been shown to correlate well with the SDQ [42]. Still, the SDQ provides a sensitivity of 82.4% and a specificity of 85.4% when a cut-off equal to the 90th percentile is chosen, as has been done in this study [28].

### Sex differences

In our study, the proportion between girls and boys having behavioural problems above the cut-off is very different (8.6 vs. 11.1%). This is expected as Smedje et al. also found these differences when validating the SDQ in a Swedish population, although the proportions of children being above the cut-off were 8.9 vs. 12.8%, respectively in their study [29]. Although it is unknown why girls seem to be more at risk in this study for behavioural problems after prenatal exposure to EDCs, previous research has shown that exposure to EDCs can lead to epigenetic changes by altering DNA methylation patterns, changing noncoding RNA expression and promoting histone modifications, which may affect neurodevelopment [43, 44]. How exactly these mechanisms affect boys and girls differently and hence, potentially lead to different behaviour remains unclear [45]. Further, specifically phenols, pesticides and phthalates are suspected to affect neurodevelopment and behaviour in a sex-dependent manner by acting through the oestrogen and androgen receptor signalling [46]. It has been shown that steroid hormones do affect neurodevelopmental disorders and an increase in testosterone might affect the mechanisms of ASD, although the latter part is not yet fully understood [45]. Explanations could lie in the potential induction of abnormal dendric growth, disruption of synaptic transmissions and receptor transmissions for neurotransmitters that can affect the neurogenesis and in turn potentially the behaviour [43, 44]. This might also, as the DNA methylation patterns above, be an explanation for different effects in girls and boys.

### Mixtures and single compounds

A study by Caporale et al. also found that mixture effects affect behaviour and delay in language acquisition [19]. Notably, the same study population (SELMA) was used and chemicals of concern in human studies were tested under experimental conditions using in vivo and in vitro models [19]. Dose-response relationships were analysed and the authors of this study found a clear link between increased exposure to the mixture of EDCs and delayed language acquisition and neurodevelopmental adversities [19]. Further, the mixture of the 20 chemicals was shown to interfere with hormonal pathways and dysregulating the expression of genes, which are linked to autism spectrum disorders [19]. This relates to findings in the current study, as the mixture analysis identified several chemicals that are of concern in all WQS analyses, while single compound analyses only showed increased associations for MBP. The similarities refer to seeing increased risks when using mixture approaches but also being able to identify some of the same chemicals of concern in the study by Caporale et al. and the current study. In general, many studies refer to mixtures as a homogenous concept as if there is a single mixture of exposure. Yet mixtures consist of a great variety of different compounds and exposure patterns that are based on personal choices and the lifestyle of exposed people [15, 16, 39]. Further, WQS regression creates a weighted index of chemicals that allows for subject-specific and different exposure patterns to contribute to the index.

### Quasipoisson vs. logistic regression

Many identified chemicals of concern were identified in both quasipoisson and logistic WQS regressions when analysing the total SDQ score compared to the cut-off at the approximated 90th percentile. A possible reason for the small difference could be that the children above the cut-off have behavioural difficulties that are clinically relevant as stated by Goodman et al. and hence, express symptoms of child psychopathology [26]. In the present study we showed that the chemicals that are of potential concern in clinical settings might also be relevant on a population level, as some of the same chemicals are of concern in quasipoisson and logistic regression models. At the same time there might be an indication that the expression of clinically relevant behavioural difficulties might be different from a small increase in the SDQ score as some other chemicals are only of concern in one of the analyses (quasipoission vs. logistic WQS regressions). Future studies should investigate if there is any difference in behavioural expression and its connection to chemicals of concern identified in this analysis.

Furthermore, Smedje et al. analysed the psychometric properties of the SDQ in Swedish setting and based on the 90th percentile, the cut-off was set at 14 (*n* = 900) [29]. In contrast, Malmberg et al. validated the SDQ for use in a Swedish setting and chose the 90th percentile cut-off at a score of 11 and higher (*n* = 263 and *n* = 230) [28]. Possible reasons for these vast differences could be differences in population characteristics. In the current study, 65.6% of mothers had a university degree, whereas 33.1% of the mothers and 22.5% of the father had a university degree in the study conducted by Malmberg et al. [28] and the study by Smedje et al. does not provide population characteristics [29]. Still, parental education, living conditions and family status could possibly influence the differences in the scores.

### Strengths and limitations

Some strengths and limitations of this study need to be considered. First, this study is based on a large ongoing pregnancy cohort that provides rich data on biological samples, background data and psychometric evaluations. Second, the outcome was assessed through a parental questionnaire (provided by psychologists at the follow up examination at 7 years of age), which potentially produces more sensitive estimates than the use of diagnoses recorded in the health registers, as most of the children are unlikely to have been diagnosed at this age, if they only present mild behavioural difficulties or have developed coping or masking strategies. Even though the parental report might have some limitations it has previously shown adequate validity [28]. Third, the repeated holdout validations improve the generalisability of the estimates, as some weights produced extreme values. This means by using 100 repeated holdout validations, we were able to generate more stable and potentially more conservative weights for most of the chemicals, as the means for the validation sample were usually lower than the means of the full sample WQS regression. This also led to all results being attenuated and only borderline significant with 94 out of 100 randomly selected holdout analyses had positive beta coefficients for girls. The generalisability of this study was improved using the 100 repeated holdout validations, but environmental hazard policies and differences in exposure levels should be taken into account when comparing findings from different countries and continents.

Taking into account the limitations, the exposure was assessed only once during pregnancy and hence, could show a skewed estimate, as we also measured short-living chemicals that are likely to be metabolised within a short amount of time. However, the majority of people are constantly exposed to short-living chemicals, which leads to a constant or omnipresent exposure and in turn leads to an approximation of the real exposure that can be compared between study participants, as the time of collection was morning for all the included study participants [47, 48]. By using creatinine levels in the urine, the estimates were corrected for urinary dilution to approximate the true value of EDC levels. Still, this could have led to a non-differential misclassification of the exposure, as this is still an approximation of the true estimates, which would likely attenuate the results towards the null, as there would be no difference in urinary dilution between those with a higher SDQ score compared to those children with a lower score. As this current study only included 26 compounds in the mixture analysis there are some unmeasured and potentially correlated compounds that study participants were exposed to that could drive the associations in any direction. Further, there may be some unmeasured confounding such as the mother’s socioeconomic status, which could have an impact on the association, although smoking is commonly used as a proxy for socioeconomic status in Sweden and has been included in the analyses [49]. Another factor that could be considered a limitation is postnatal exposure to EDCs. While the postnatal period is still sensitive, we only considered prenatal exposure of EDCs in this study. As the neurogenesis starts early in pregnancy as described above, it might be the most important time to assess exposure for neurodevelopmental outcomes, but postnatal exposure could still drive the association in any direction. As is the case for all mixture analysis studies, inference from our results is limited to prenatal exposures with similar exposure patterns. However, Sapounidou et al. found that 66% of women of reproductive age in the US (NHANES data) have sufficiently similar exposure patterns and chemicals of concern to women included in our study [50].

## Conclusion

We were able to detect a statistically significant increase in the total SDQ score for girls, in connection to exposure to mixtures of EDCs, that also seems to be of importance after applying 100 repeated holdout validations to be able to generalise the results to other populations. Chemicals of concern were non-persistent and mainly plasticizers. These chemicals were identified both when a continuous metric of behaviour (quasipoisson) and clinically relevant cut-offs (logistic) were used. Future research should focus on how biological mechanisms affect girls and boys differently when exposed to EDCs.

## Supplementary information

Supplementary material

## Data Availability

These data cannot be made publicly available. According to the General Data Protection Regulation, the Swedish law, the Swedish Data Protection Act, the Swedish Ethical Review Act, and the Public Access to Information and Secrecy Act, these types of sensitive data can only be made available for specific purposes, including research, that meets the criteria for access to this type of sensitive and confidential data as determined by a legal review. For more information about the data, please contact Carl-Gustaf.Bornehag@kau.se
