# Supplementary material for: Prenatal exposure to endocrine disrupting chemicals and the association with behavioural difficulties in 7-year-old children in the SELMA study
Source: J Expo Sci Environ Epidemiol. 2024 Dec 19;35(6):981–91. doi: 10.1038/s41370-024-00739-x (PMC12583134; doi:10.1038/s41370-024-00739-x)
Supplement: Supplementary file 1 — Supplementary material [file 41370_2024_739_MOESM1_ESM.docx]

SUPPLEMENTARY MATERIAL

Supplementary table 1: Differences between characteristics in included and excluded study population

|  | Included study population | | Excluded study population | | P-value ^b^ |
| --- | --- | --- | --- | --- | --- |
|  | n = 607 | | n = 354 ^c^ | |  |
| Maternal characteristics |  |  |  |  |  |
| Parity (n, %) |  |  |  |  |  |
| *1^st^ child* | 319 | 52.6% | 141 | 39.8% | 0.08 |
| *Missing* | 0 |  | 14 | 4.0% |  |
| Tobacco exposure ^a^ (n, %) |  |  |  |  |  |
| *Yes* | 58 | 9.6% | 44 | 12.4% | 0.17 |
| *Missing* | 0 |  | 5 | 1.4% |  |
| Graduated university (n, %) |  |  |  |  |  |
| *Yes* | 398 | 65.6% | 194 | 54.8% | 0.03 |
| *Missing* | 0 |  | 20 | 5.6% |  |
| Age at birth (mean, SD) | 31.0 | 4.4 | 30.9 | 5.1 | 0.70 |
| *Missing* | 0 |  | 21 |  |  |
| BMI (mean, SD) | 24.7 | 4.5 | 28.5 | 24.0 | 0.02 |
| *Missing* | 0 |  | 148 |  |  |
| Child characteristics |  |  |  |  |  |
| Age at outcome assessment (mean, SD) | 7.5 | 0.3 | 7.6 | 0.3 | 0.03 |
| *Missing* | 0 |  | 3 |  |  |
| SDQ score (median, IQR) | 5.5 | 4.4 | 6.4 | 5.2 | 0.02 |
| *Missing* | 0 |  | 64 |  |  |
| Above the 90^th^ percentile approximation for SDQ score (n, %) | 60 | 9.9% | 38 | 10.7% | 0.18 |
| *Missing* | 0 |  | 64 |  |  |
| ^a^ Tobacco exposure based on questionnaire if the mother, father or another person in the household smoked or serum cotinine levels ≥ 0.2 ng/ mL  ^b^ p-values were calculated using students t-test for continuous variables and chi-square tests were used for categorical variables. The p-value for the SDQ score was calculated using the Wilcoxon rank sum test due to a skewed distribution  ^c^ Not all 354 participants with missing data are shown here as some also had missing data on the exposure measurements. | | | | | |

Supplementary table 2: Summary of chemicals of concern in different mixture analysis

|  | **Stratified analysis** | | | |
| --- | --- | --- | --- | --- |
|  | **Girls** | | **Boys** | |
|  | Quasipoisson | Logistic | Quasipoisson | Logistic |
| **Chemicals** |  |  |  |  |
| Triclosan | Possible |  |  |  |
| BPA |  |  |  | Possible |
| BPF | Possible |  | Possible | Possible |
| BPS |  |  |  | Possible |
| MEP |  |  |  |  |
| MBP | Possible * | Possible |  |  |
| MBzP | Possible | Possible | Possible | Possible |
| DEHP |  | Possible |  |  |
| DINP |  |  |  |  |
| MHiDP |  |  |  |  |
| MCiNP |  |  |  |  |
| MOiNCH |  |  | Possible | Possible |
| DPHP | Possible | Possible |  |  |
| TCP | Possible |  | Possible | Possible |
| PBA |  | Possible | Possible | Possible |
| 2OHPH |  | Possible |  |  |
| PFOA |  |  |  |  |
| PFOS |  |  |  |  |
| PFNA |  |  |  |  |
| PFDA |  |  |  |  |
| PFUnDA |  |  |  |  |
| PFHxS | Possible |  |  |  |
| HCB |  |  |  |  |
| Nonachlor |  |  |  |  |
| DDT |  |  |  |  |
| PCB |  |  |  |  |
| * refers to chemicals in single compound analysis that showed significant adverse associations Possible: Chemicals with possible contribution to the mixture effect, between 50 and 90% of repetitions were above the threshold for chemicals of concern. | | | | |

Supplementary table 3: Percentages for weights of chemicals of concern in validation sets analyses, identified in both full and validation sample analyses, based on figures 1-4 in main manuscript

|  | Stratified analysis | | | |
| --- | --- | --- | --- | --- |
|  | Girls (n = 302) | | Boys (n = 305) | |
| Chemicals | Quasipoisson | Logistic | Quasipoisson | Logistic |
| Triclosan | 6 | 5 |  |  |
| BPA |  |  | 5 | 12 |
| BPF | 5 |  | 11 | 8 |
| BPS |  |  | 5 | 6 |
| MEP | 5 | 5 |  |  |
| MBP | 14 | 6 | 5 |  |
| MBzP | 8 | 7 | 11 | 7 |
| DEHP | 5 | 10 |  |  |
| DINP |  |  |  |  |
| MHiDP |  |  |  |  |
| MCiNP |  | 5 |  |  |
| MOiNCH |  |  | 6 | 7 |
| DPHP | 7 | 8 | 5 |  |
| TCP | 10 |  | 11 | 6 |
| PBA |  |  | 8 | 8 |
| 2OHPH | 4 | 8 |  | 5 |
| PFOA |  |  | 4 | 5 |
| PFOS |  |  |  |  |
| PFNA |  |  |  |  |
| PFDA |  |  |  |  |
| PFUnDA |  |  |  | 5 |
| PFHxS | 5 | 4 |  |  |
| HCB |  |  |  |  |
| Nonachlor |  | 4 |  |  |
| DDT |  |  |  |  |
| PCB |  |  |  | 4 |
| ^a^ The first number is the weight for the full sample analysis and the number in the parenthesis is the weight for the validation sample analysis | | | | |

Supplementary table 4: Associations between the mixture of 26 chemicals and continuous SDQ score estimated by quasipoisson regression using betas and 95% CI and the dichotomised SDQ score with a 90^th^ percentile cut-off approximation estimated by logistic regression using OR and 95% CI with birthweight and gestational age added as potential mediators and maternal IQ as potential confounder (n = 597)

|  | *Main analysis ^a^* | | *Birthweight*  ^b^ | | *Gestational age* ^c^ | | *Maternal IQ* ^d^ | | *Gestational age, birthweight and maternal*   *IQ ^e^* | |
| --- | --- | --- | --- | --- | --- | --- | --- | --- | --- | --- |
|  | n = 607 | | n = 597 | | n = 597 | | n = 597 | | n = 597 | |
|  | Estimate | 95% CI | Estimate | 95% CI | Estimate | 95% CI | Estimate | 95% CI | Estimate | 95% CI |
| Quasipoisson WQS ( β ) | 0.09 | 0.04, 0.15 | -0.02 | -0.09, 0.04 | 0.09 | 0.04, 0.14 | 0.09 | 0.04, 0.15 | -0.02 | -0.09, 0.04 |
| Logistic WQS (OR) | 1.35 | 1.05, 1.74 | 1.07 | 0.79, 1.44 | 1.39 | 1.07, 1.81 | 1.39 | 1.08, 1.81 | 1.07 | 0.80, 1.44 |
| a Adjusted for parity, maternal tobacco exposure, maternal education, maternal age, maternal BMI, child sex and child’s age at outcome assessment and creatinine  ^b^ Adjusted for parity, maternal tobacco exposure, maternal education, maternal age, maternal BMI, child sex and child’s age at outcome assessment and birthweight and creatinine  ^c^ Adjusted for parity, maternal tobacco exposure, maternal education, maternal age, maternal BMI, child sex and child’s age at outcome assessment and gestational age and creatinine  ^d^ Adjusted for parity, maternal tobacco exposure, maternal education, maternal age, maternal BMI, child sex and child’s age at outcome assessment and maternal IQ and creatinine  ^e^ Adjusted for parity, maternal tobacco exposure, maternal education, maternal age, maternal BMI, child sex and child’s age at outcome assessment, birthweight, gestational age and maternal IQ and creatinine | | | | | | | | | | |

Supplementary table 5: Association between the mixture of 26 chemicals and continuous SDQ estimated by quasipoisson regression using quartiles (n = 607)

|  | Adjusted ^a, b^ | | Stratified ^c^ | | | | Interaction ^d^ |
| --- | --- | --- | --- | --- | --- | --- | --- |
|  | All children (n = 607) | | Girls (n = 302) | | Boys (n = 305) | |  |
|  | Estimate | 95% CI | Estimate | 95% CI | Estimate | 95% CI |  |
| **Full sample ^e^** |  |  |  |  |  |  |  |
| Deciles  (main analysis) | 0.09 | 0.04, 0.15 | 0.12 | -0.02, 0.27 | 0.09 | 0.02, 0.16 | p = 0.36 |
| Quartiles  (sensitivity analysis) | 0.25 | 0.11, 0.39 | 0.36 | -0.02, 0.76 | 0.28 | 0.09, 0.44 | p = 0.35 |
| **Validation ^f^** |  |  |  |  |  |  |  |
| Deciles | 0.03 | -0.03, 0.08 | 0.03 | -0.03, 0.10 | -0.01 | -0.08, 0.06 | p = 0.25 |
| Quartiles | 0.07 | -0.06, 0.21 | 0.17 | -0.07, 0.42 | 0.00 | -0.19, 0.20 | p = 0.26 |
| ^a^ Adjusted for parity, maternal tobacco exposure, maternal education, maternal BMI, maternal age, child sex and child’s age at outcome assessment and creatinine  ^b^ Adjusted for parity, maternal tobacco exposure, maternal education, maternal age, maternal BMI and child’s age at outcome assessment and creatinine  ^c^ Interaction between the mixture of EDCs and sex  ^d^ WQS regression using all 607 children in a single quasipoisson or logistic model  ^fe^100 repeated holdout validations using 40% of the data as training and 60% as validation data | | | | | | |  |

**
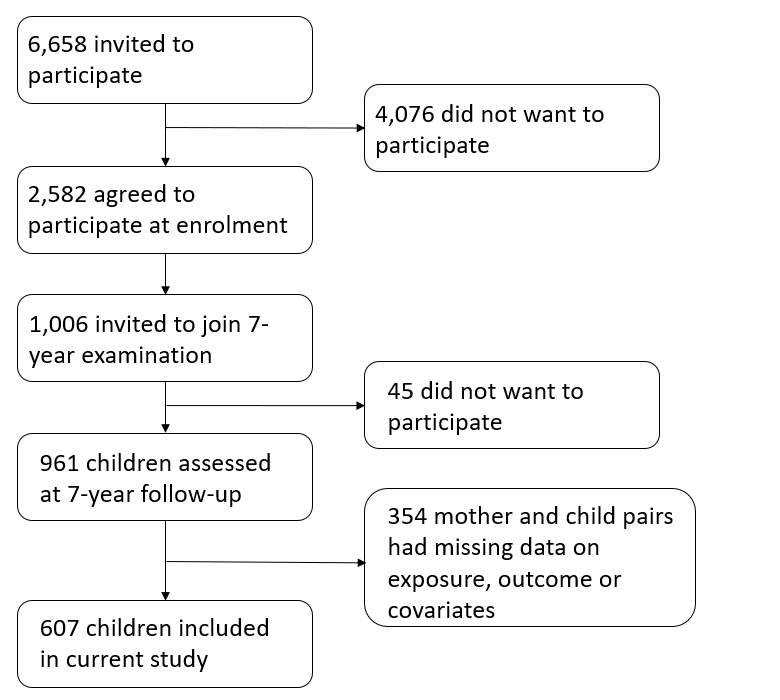
**

Supplementary figure 1: Flowchart of the study population

**
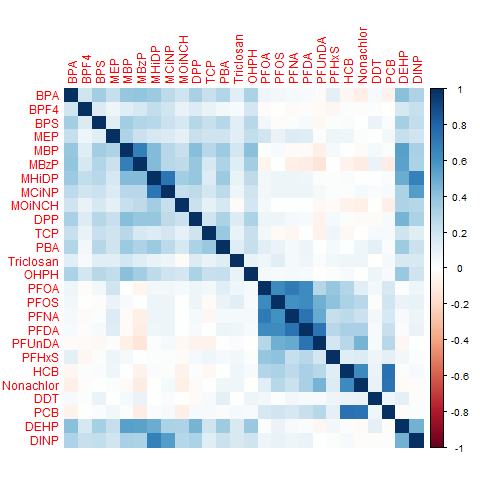
**

Supplementary figure 2: Correlation matrix for all included 26 chemicals


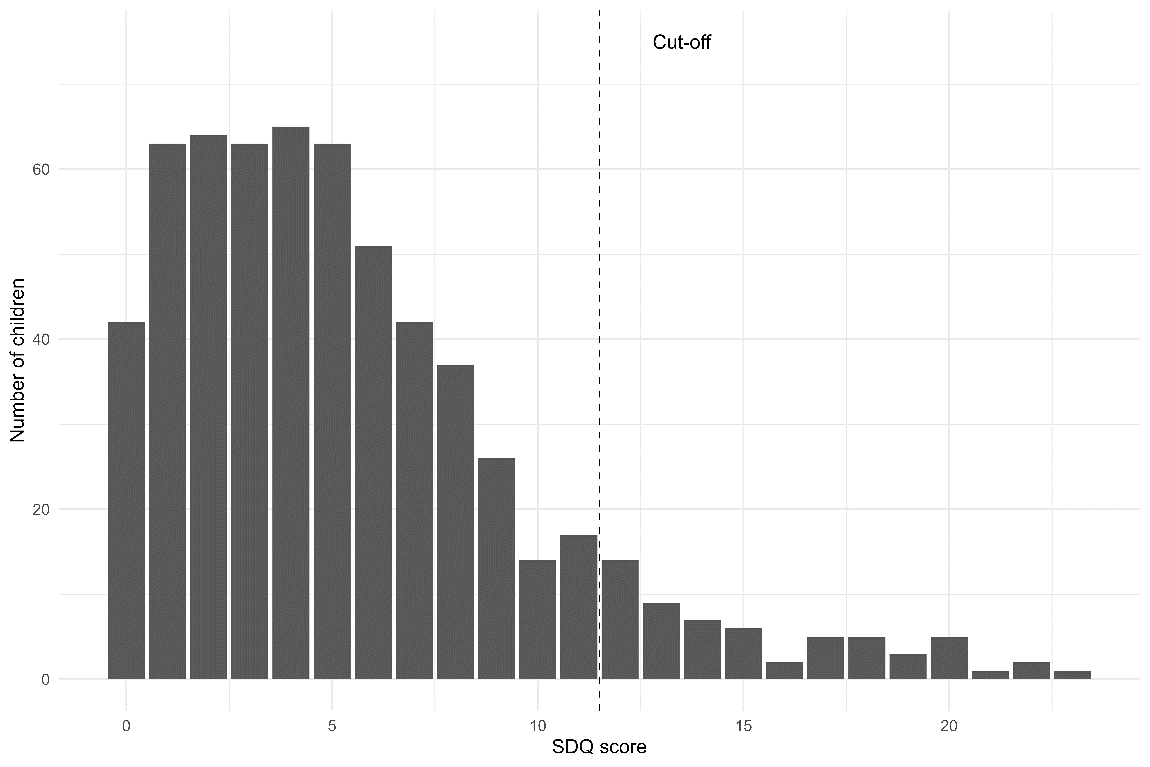


Supplementary figure 3: Distribution of the outcome variable (SDQ score) with the indicated cut-off for clinical cases above a score of 11.

**
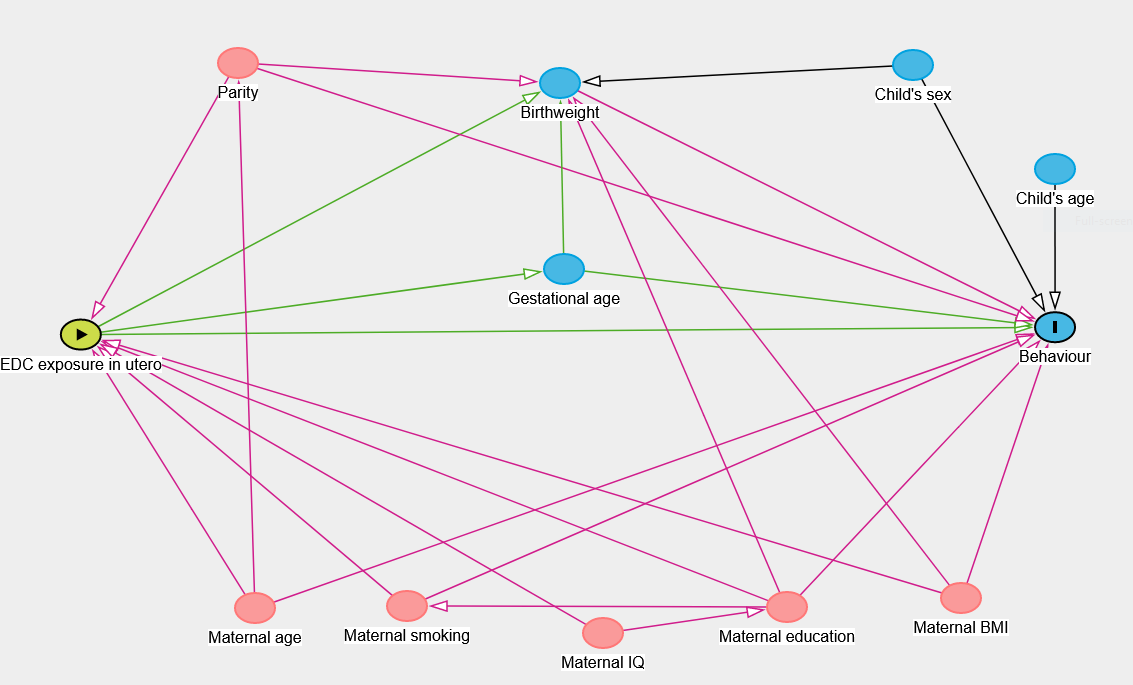
**

Supplementary figure 4: directed acyclic graph for the association between EDC exposure in utero and behaviour in children.


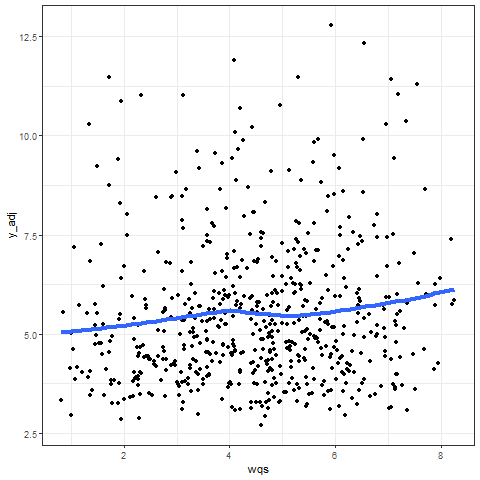


Supplementary figure 5: Loess curve testing for non-linearity overlaid on a scatterplot for the WQS index and the adjusted continuous SDQ.


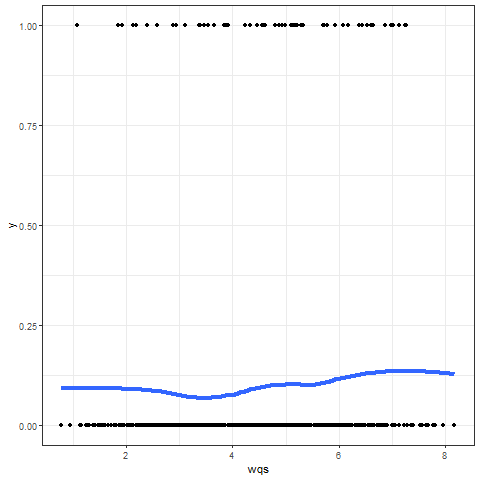


Supplementary figure 6: Loess curve testing for non-linearity overlaid on a scatterplot for the WQS index and the binary SDQ.


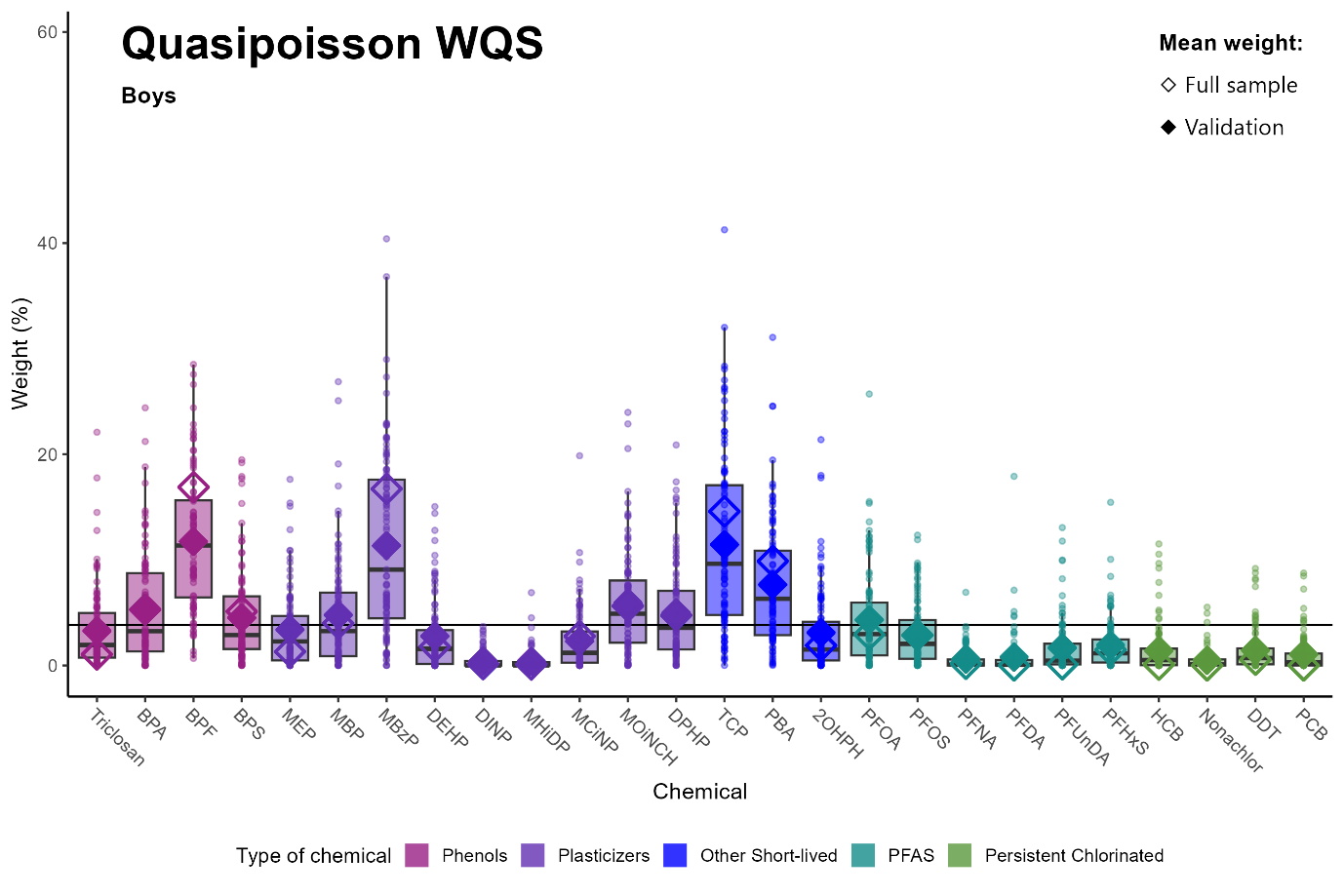


Notes: Data points indicate weights for each of the 100 holdouts. Box plots show 25^th^, 50^th^ and 75^th^ percentiles and whiskers show 10^th^ and 90^th^ percentiles of weights for the 100 holdouts. Closed diamonds show mean weights for the 100 holdouts and open diamonds show the mean weight of the full sample analysis. The black line at 3.84% indicates the threshold of chemicals of concern.
Models were adjusted parity, maternal tobacco exposure, maternal education, maternal age, maternal BMI, child sex and child’s age at outcome assessment and creatinine.

Supplementary figure 7: Identification of chemicals of concern and uncertainty using traditional full sample vs. repeated holdout validation: adjusted quasipoisson WQS for boys (n = 305)


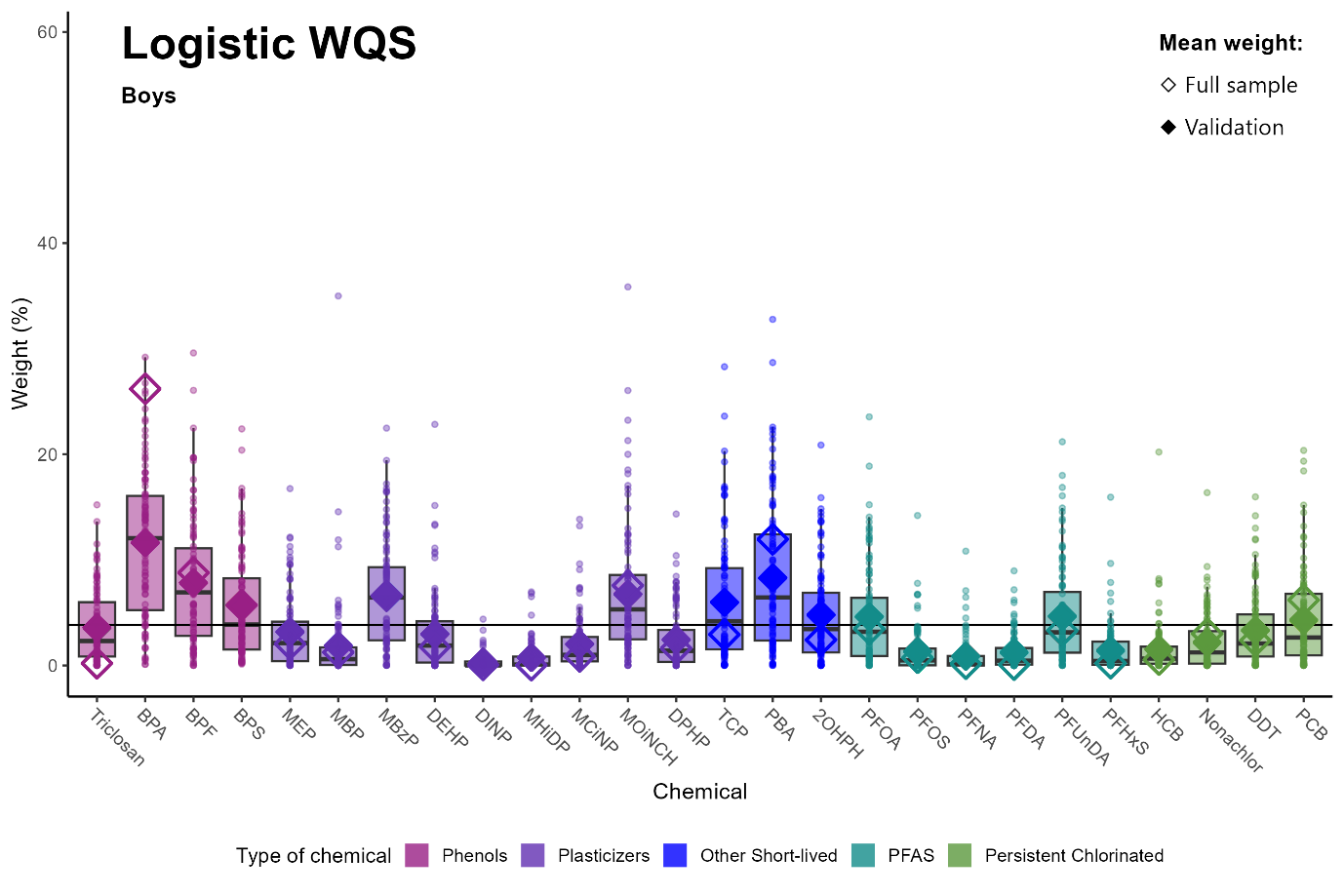


Notes: Data points indicate weights for each of the 100 holdouts. Box plots show 25^th^, 50^th^ and 75^th^ percentiles and whiskers show 10^th^ and 90^th^ percentiles of weights for the 100 holdouts. Closed diamonds show mean weights for the 100 holdouts and open diamonds show the mean weight of the full sample analysis. The black line at 3.84% indicates the threshold of chemicals of concern.
Models were adjusted parity, maternal tobacco exposure, maternal education, maternal age, maternal BMI, child sex and child’s age at outcome assessment and creatinine.

Supplementary figure 8: Identification of chemicals of concern and uncertainty using traditional full sample vs. repeated holdout validation: adjusted logistic WQS for boys (n = 305)


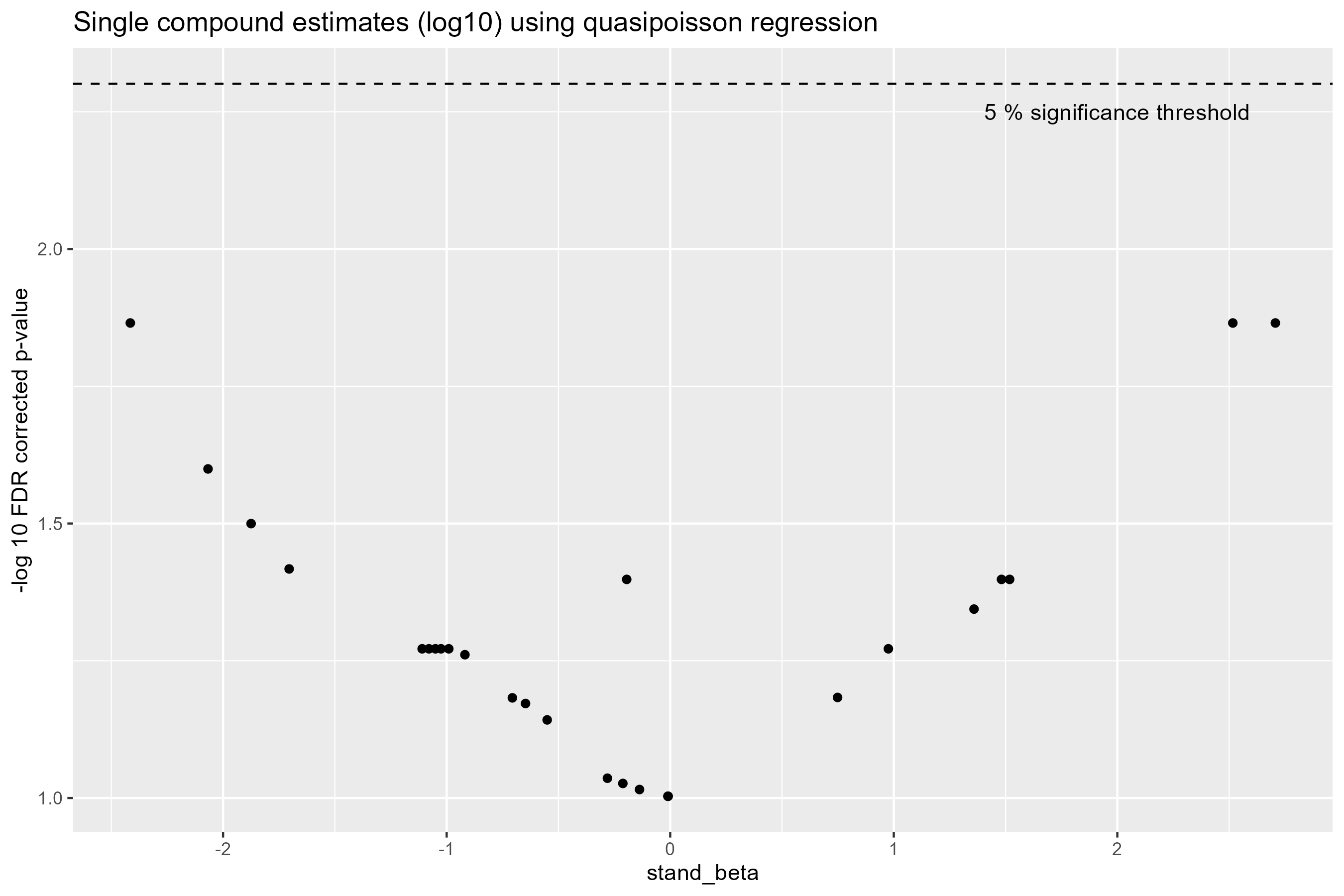
Supplementary figure 9: Volcano plot showing the multiple testing corrected associations between single compounds (ng/ml, centered and scaled) and the continuous SDQ score by quasipoisson regression for all children (n = 607)

Note: All models for urinary compounds are adjusted for urinary creatinine concentration.
Adjusted for parity, maternal tobacco exposure, maternal education, BMI, maternal age and child’s age at outcome assessment and sex.

Supplementary figure 10: Volcano plot showing the multiple testing corrected associations between single compounds (ng/ml, centered and scaled) and the continuous SDQ score by quasipoisson regression for girls (n = 302)


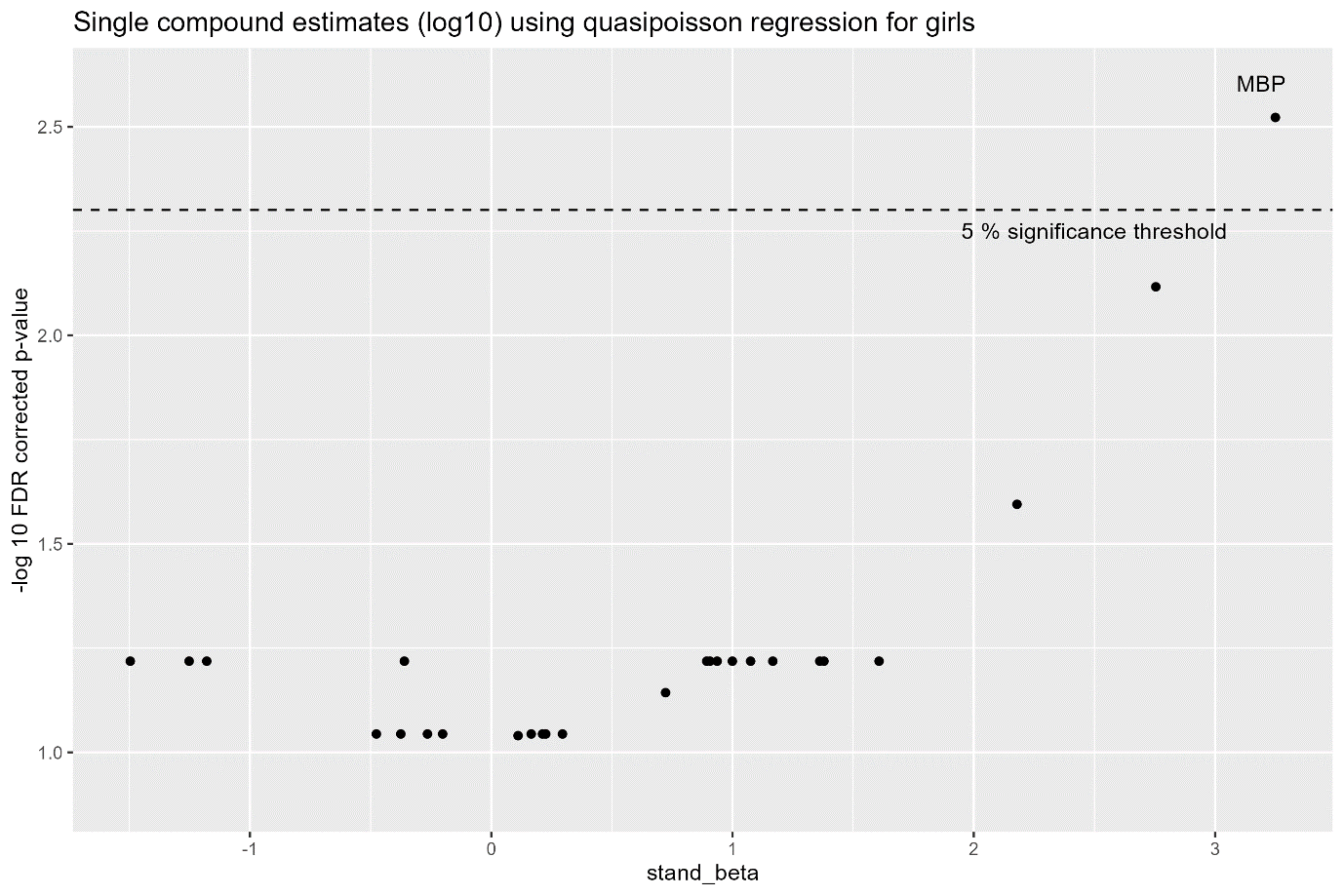


Note: All models for urinary compounds are adjusted for urinary creatinine concentration.
Adjusted for parity, maternal tobacco exposure, maternal education, BMI, maternal age and child’s age at outcome assessment.

Supplementary figure 11: Volcano plot showing the multiple testing corrected associations between single compounds (ng/ml, centered and scaled) and the continuous SDQ score by quasipoisson regression for boys (n = 305)


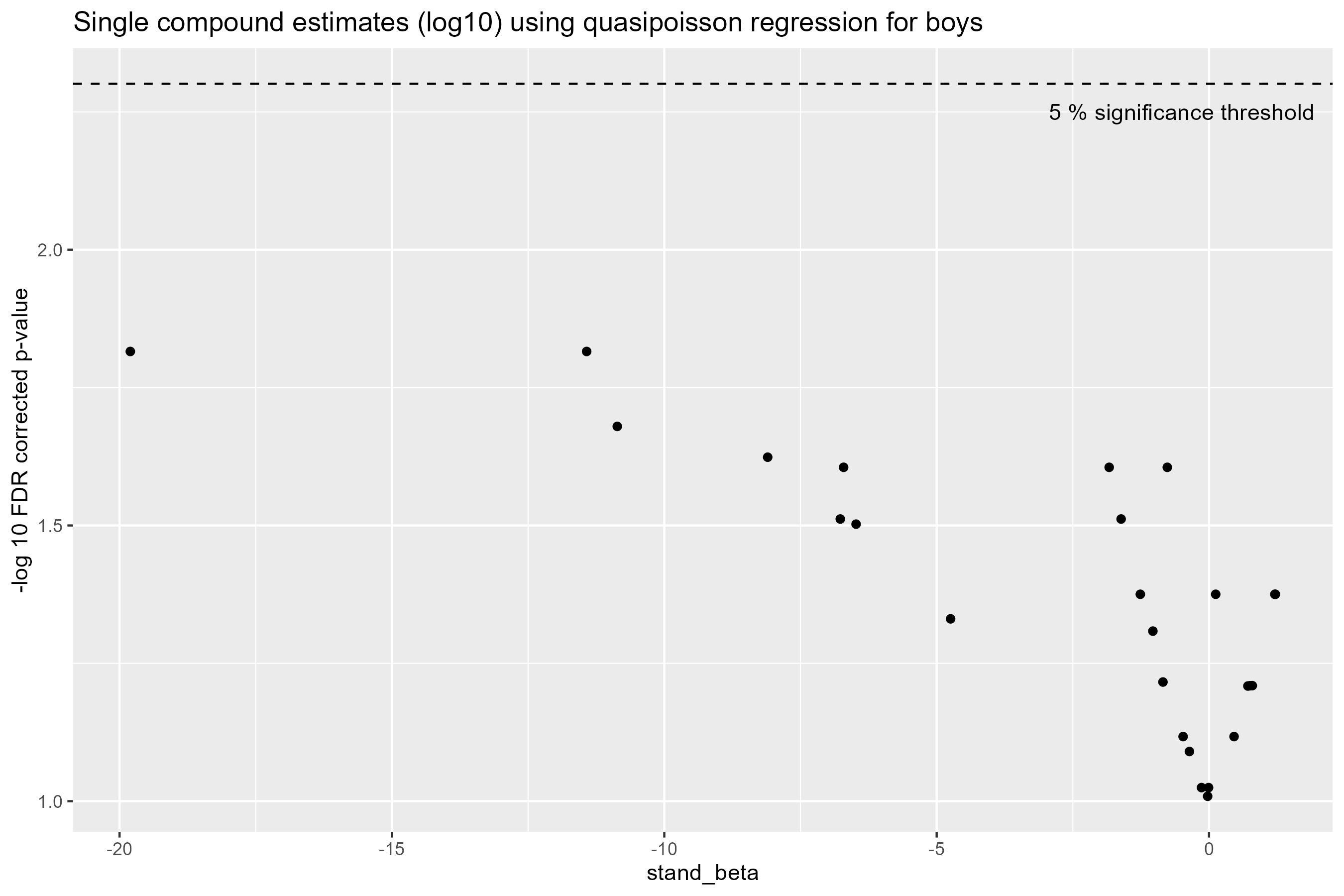


Note: All models for urinary compounds are adjusted for urinary creatinine concentration.
Adjusted for parity, maternal tobacco exposure, maternal education, BMI, maternal age and child’s age at outcome assessment.

Supplementary figure 12: Volcano plot showing the multiple testing corrected associations between single compounds (ng/ml, centered and scaled) and the dichotomous SDQ score by logistic regression for all children (n = 607)


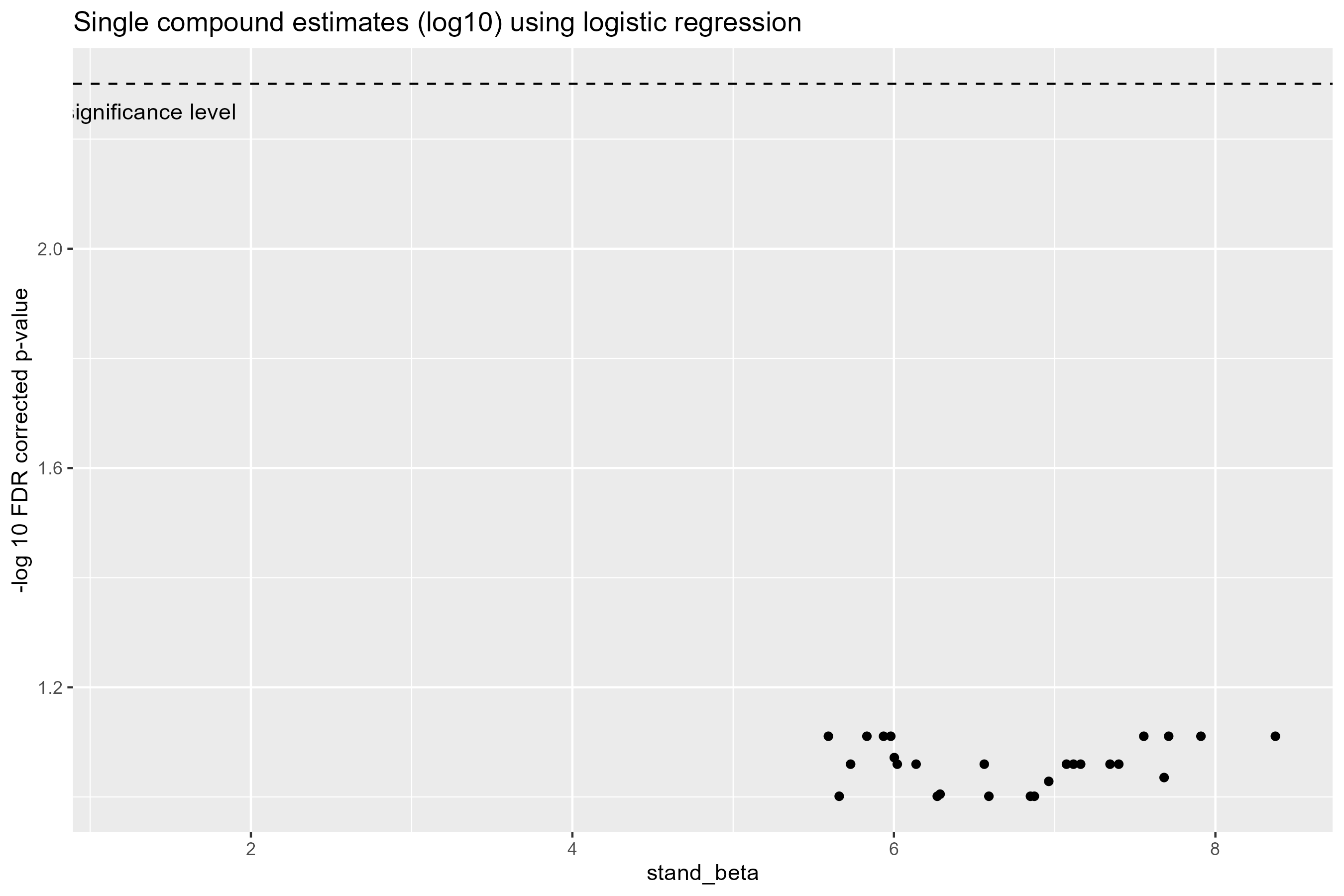


Note: All models for urinary compounds are adjusted for urinary creatinine concentration.
Adjusted for parity, maternal tobacco exposure, maternal education, BMI, maternal age and child’s age at outcome assessment and sex.

Supplementary figure 13: Volcano plot showing the multiple testing corrected associations between single compounds (ng/ml, centered and scaled) and the dichotomous SDQ score by logistic regression for girls (n = 302)


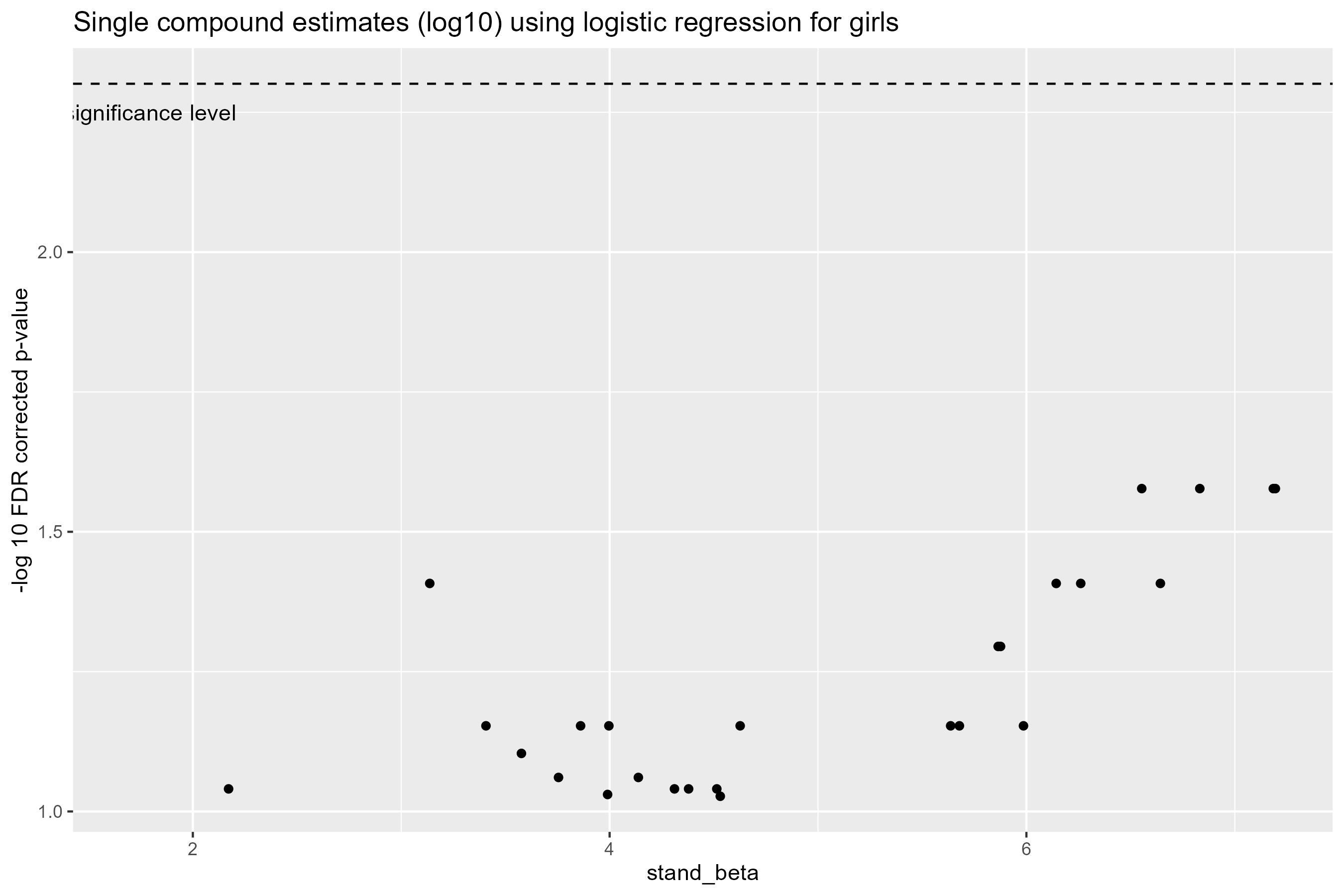


Note: All models for urinary compounds are adjusted for urinary creatinine concentration.
Adjusted for parity, maternal tobacco exposure, maternal education, BMI, maternal age and child’s age at outcome assessment.

Supplementary figure 14: Volcano plot showing the multiple testing corrected associations between single compounds (ng/ml, centered and scaled) and the dichotomous SDQ score by logistic regression for boys (n = 305)
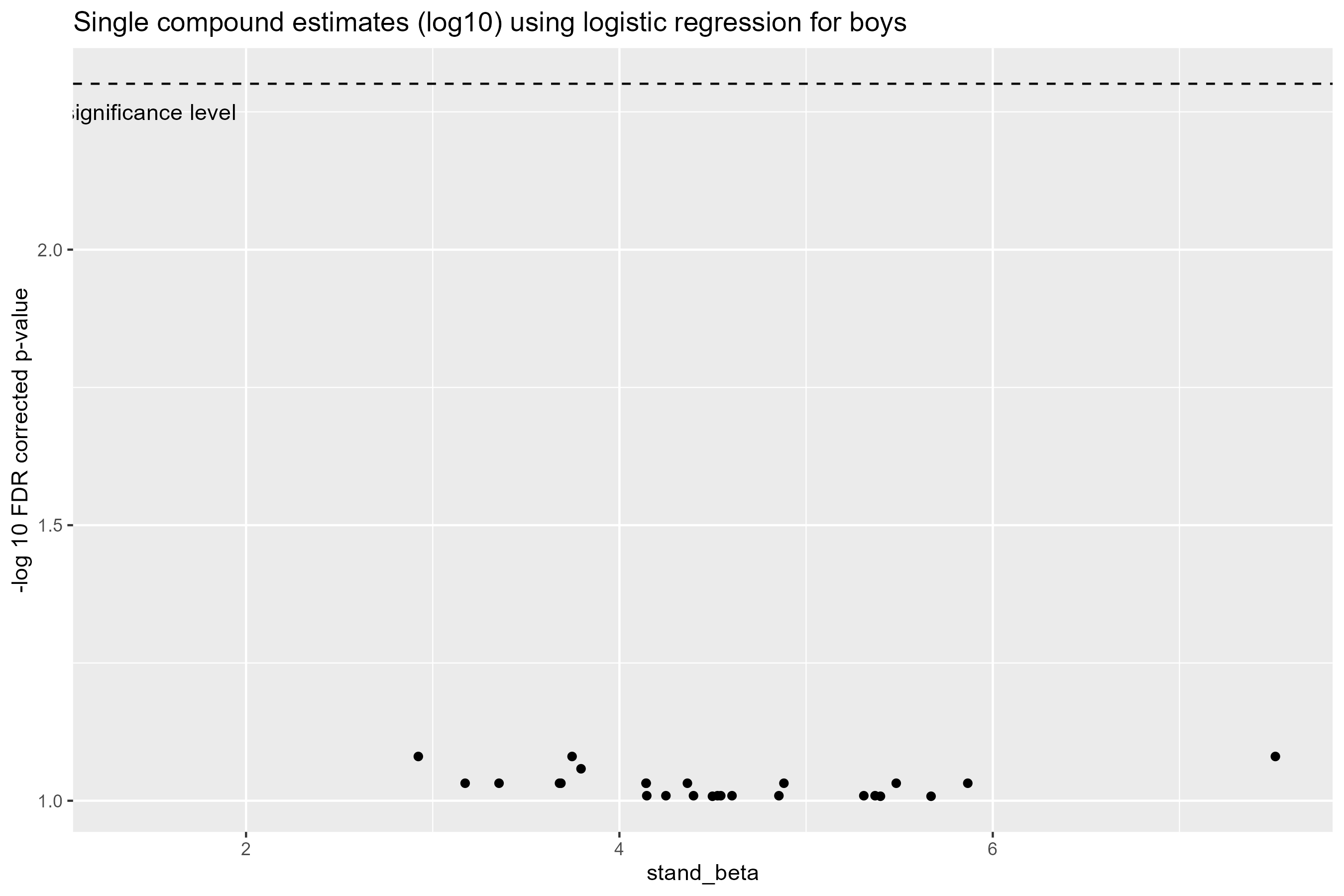


Note: All models for urinary compounds are adjusted for urinary creatinine concentration.
Adjusted for parity, maternal tobacco exposure, maternal education, BMI, maternal age and child’s age at outcome assessment.
